# Association of atopic dermatitis with obesity via a multi-omics approach: A protocol for a case–control study

**DOI:** 10.1097/MD.0000000000016527

**Published:** 2019-07-19

**Authors:** Mi Ju Son, Geum-Jin Yang, Eun-Heui Jo, Yu-Hwa Shim, Su-Jin Kang, Ji-Eun Hong, Young-Eun Kim, Jung-Eun Lee, Jaemoo Chun, Seonghwan Park, Jeeyoun Jung, Min-Cheol Park

**Affiliations:** a Clinical Medicine Division, Korea Institute of Oriental Medicine, Daejeon; b Korean Medicine Dermatology Clinical Research Center of Wonkwang University, Iksan; c Department of Acupuncture and Moxibustion, Jeonju Hospital of Oriental Medicine of Wonkwang University, Jeonju; d Department of Korean Medicine Obstetrics & Gynecology, Wonkwang University Iksan Korean Medicine Hospital, Iksan; e Future Medicine Division, Korea Institute of Oriental Medicine, Daejeon; f Department of Korean Medicine Ophthalmology and Otolaryngology and Dermatology, Wonkwang University Iksan Korean Medicine Hospital, Iksan, Republic of Korea.

**Keywords:** atopic dermatitis, case–control study, immune biomarker, metabolome, microbiome, obesity

## Abstract

**Introduction:**

Several studies have found that obesity is associated with atopic dermatitis (AD); however, the mechanisms underlying the association are largely unknown. This study aims to assess the association of AD with obesity in the Korean population and verify its mechanism via a multi-omics analysis.

**Methods and analysis:**

A case–control study will be conducted in the Republic of Korea. A total of 80 subjects, aged 4 to 12 years, matched for age and sex, with body mass index at or above the 85th percentile or at or below the 25th percentile, will be included. Subjects will be assigned to the following 4 groups: obese/overweight with AD, normal/underweight with AD, obese/overweight control, and normal/underweight control. Serum metabolome and immune biomarkers, as well as fecal metabolome and microbiome biomarkers, will be analyzed. Serum eosinophil cationic protein, total serum Immunoglobulin E (IgE), and specific IgE will be analyzed to assess allergic tendency. The SCORing of AD index, the children's dermatology life quality index, body composition analysis, and the Korean gastrointestinal symptom rating scale will be obtained to assess the disease status and severity of the subjects.

**Discussion:**

The findings of this study are expected to provide evidence of an association between AD and obesity via a gut microbiome-metabolome-immune mechanism. Therefore, it may improve future management strategies for AD.

**Trial registration:**

This study has been registered at the Korean National Clinical Trial Registry, Clinical Research Information Service (KCT0003630).

## 1 Introduction

Atopic dermatitis (AD), also known as atopic eczema, is a common chronic inflammatory skin disease characterized by extensive pruritus, a clinical course of symptomatic flares and recurrent eczematous lesions.^[1]^ The pathophysiology of AD is complex and multifactorial, involving epidermal barrier disruption, skin inflammation due to dysregulation of immune responses, immunoglobulin E (IgE)-mediated hypersensitivity, and environmental factors.^[2]^ Although the direct cause and pathophysiology of AD have not been clearly elucidated, several studies have suggested that obesity is associated with AD. Recent systematic reviews found that being overweight or obese was associated with increased prevalence and risk of AD.^[3–5]^ Moreover, levels of serum leptin, a polypeptide hormone produced by adipocytes that helps regulate energy balance by inhibiting hunger,^[6]^ decreased as AD became severe.^[7]^ However, the mechanisms underlying this association are largely unknown.

The gut microbiome has attracted much attention beyond classical infectious diseases. Numerous studies have reported changes in the gut microbiota, not only in gastrointestinal disease but also in obesity and allergic diseases.^[8,9]^ Recent studies have indicated that dysbiosis in *Faecalibacterium prausnitzii* and dysregulation of gut epithelial inflammation might affect the chronic progression of AD by resulting in impairment of the gut epithelial barrier.^[10]^ Alterations in the proportions of *Bacteroidetes* and *Firmicutes*—which account for the majority of gut microbiota—lead to obesity, associated with relatively low gut microbial diversity.^[11,12]^

Based on these previous studies, we hypothesize that the gut microbiome is involved in the mechanism of AD and obesity. This study aims to assess the association between obesity and AD in the context of gut microbiome by analyzing the gut microbiome, the metabolome, and immune biomarkers.

## 2 Methods

### 2.1 Objectives

The aim of this study is to assess the association of AD with obesity in the Korean population and verify its mechanism by analyzing gut microbiome–metabolome–immune biomarkers using a multi-omics approach.

### 2.2 Study design and procedure

This case-control study will be conducted at Wonkwang University Iksan Korean Medicine Hospital in the Republic of Korea. The study has 4 arms: an obese/overweight with AD group, a normal/underweight with AD group, an obese/overweight control group, and a normal/underweight control group. Twenty subjects will be assigned to each arm and a total of 80 subjects will be recruited for this study.

After potential participants voluntarily consent to the study, they will be screened using predetermined inclusion/exclusion criteria at the first visit. To be assigned to a group, subjects will be evaluated based on demographic characteristics, medical history, and physical examination including Hanifin & Rajka AD criteria. The SCORing of AD (SCORAD) index, the Children's Dermatology Life Quality Index (CDLQI), and the Korean Gastrointestinal Symptom Rating Scale (KGSRS); body composition analysis will also be performed to confirm the subjects’ general health and disease status.

The 3-day controlled meal will be provided to the subjects in order to reduce microbial changes due to variable food intake. The 3-day meal is prepared in an allergy-free manufacturing facility and is free of eggs, milk, soy, and wheat, which are known to be the main causes of food allergies.

**Figure 1:**
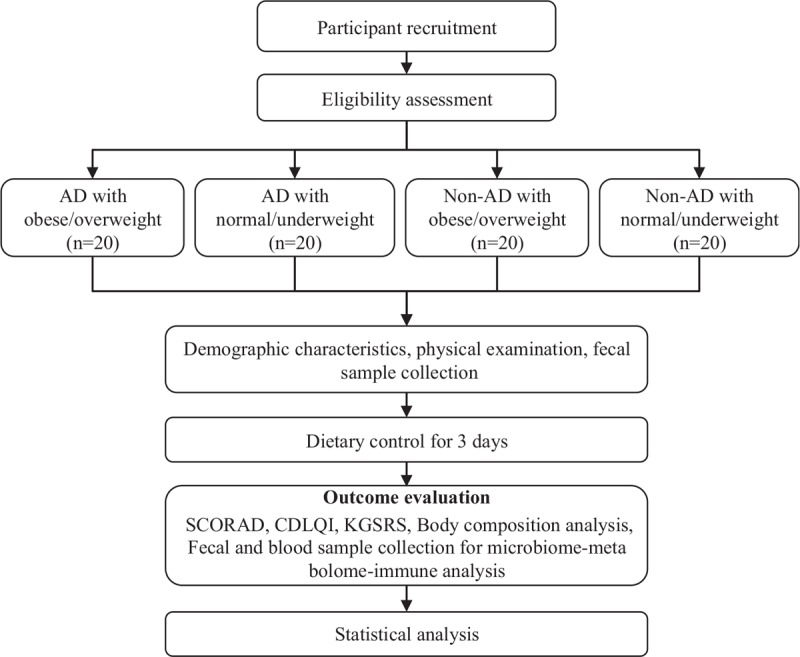
Flowchart describing the study plan. AD = atopic dermatitis; CDLQI = Children's Dermatology Life Quality Index; KGSRS = Korean Gastrointestinal Symptom Rating Scale; SCORAD = Scoring of Atopic Dermatitis.

After the 3-day meal, SCORAD index, CDLQI, KGSRS, and body composition analysis will be evaluated. Blood and fecal samples will be obtained after the controlled meal in order to analyze microbiome-metabolome-immune biomarkers. A fecal sample will also be obtained before the controlled meal in order to assess the microbial differences between the experimental groups before and after the controlled meal. The detailed design is summarized in Figure 1.

### 2.3 Participants

Participants defined as having AD are those who satisfy the Hanifin & Rajka AD criteria. Non-AD subjects are those who do not satisfy the Hanifin & Rajka AD criteria and not have asthma, allergic rhinitis, allergic conjunctivitis, or food allergies.

The obese/overweight subjects are those with a body mass index (BMI) at or above the 85th percentile, matched for age and sex. The normal/underweight patients have a BMI at or below the 25th percentile, matched for age and sex. BMI percentile in the same age and sex group is decided based on the World Health Organization (WHO) Child Growth Standards proposed in 2007.

#### 2.3.1 Inclusion criteria

1. Boys or girls aged 4 to 12 years old.
2. BMI at or above the 85th percentile, or at or below the 25th percentile, matched for age and sex based on the WHO Child Growth Standards proposed in 2007.
3. Subjects who can follow the dietary guidelines for 3 days.
4. Subjects who agree to participate and voluntarily sign a written informed consent form from the legal representatives and/or participants.

#### 2.3.2 Exclusion criteria

1. Severe skin disease or systemic disease.
2. Use of oral antibiotics, corticosteroids, anti-histamines, or immunosuppressive drugs within the last 4 weeks.
3. Use of prebiotics, probiotics, or herbal medicine within the last 4 weeks.
4. Expectation of abnormal defecation during the study period because of constipation.
5. Any subject considered unsuitable for participation in the study by the investigator.

#### 2.3.3 Recruitment

Subject recruitment began in December 2018 at Wonkwang University Iksan Korean Medicine Hospital and is expected to end by September 2020. The study will be advertised through the hospital, community residences, and local landmarks bulletin boards.

### 2.4 Variables

#### 2.4.1 Microbiome analysis

Microbiome analysis will be conducted from fecal samples. The deoxyribonucleic acid (DNA) in the collected fecal samples are isolated using the FastDNA SPIN kit (MPBio). PCR will be performed using a primer to amplify the V3–V4 gene region of 16S ribosomal ribonucleic acid (rRNA) from the separated DNA. After purification of the amplified product, a genomic DNA library will be constructed and the nucleotide sequence analyzed.

#### 2.4.2 Metabolite profiling

Nuclear magnetic resonance spectroscopy and mass spectrometry will be used to systematically identify and quantify the movement and secretion changes of metabolites in the blood.

#### 2.4.3 Immune biomarker analysis

Multiplex immunoassay will be used to analyze the serum Th1/Th2 cytokines including interleukin (IL)-2, IL-4, IL-6, IL-8, tumor necrosis factor (TNF)-α, interferon (IFN)-ɣ, and so on. The total IgE and eosinophil cationic protein (ECP) in the serum will be analyzed. The specific IgE levels for the following 12 specific antigens will be measured using the ImmunoCAP assay: egg white, milk, walnut, wheat, peanut, soy, shrimp, buckwheat, fish (cod), meat mixture, *Dermatophagoides pteronyssinus*, and *Dermatophagoides farinae*.

#### 2.4.4 Questionnaires

Questionnaires validated for each purpose and modified to a Korean version will be utilized to measure skin status and its impact on quality of life. The SCORAD index^[13]^ will be used to measure AD severity and the CDLQI^[14]^ will be used to assess the impact of skin disease on the quality of life. The KGSRS^[15]^ will also be examined to assess the severity and frequency of subjects’ gastrointestinal symptoms.

#### 2.4.5 Body composition analysis

The body composition test quantitatively analyzes the components of the human body including body water, proteins, minerals, and body fat, to assess the health and obesity status. These factors will be measured by a body composition analyzer (Inbody 3.0; Biospace, Seoul, Republic of Korea).

#### 2.4.6 Laboratory analysis

To check subjects’ health status, the following parameters will be analyzed in blood samples: white blood cell (WBC) and differential count (neutrophil, lymphocytes, monocytes, eosinophils, and basophils), red blood cell (RBC), hemoglobin, hematocrit, platelets, mean corpuscular volume (MCV), mean corpuscular hemoglobin (MCH), mean corpuscular hemoglobin concentration (MCHC), erythrocyte sedimentation rate (ESR), hemoglobin A1c, serum glucose-fasting, total cholesterol, high density lipoprotein (HDL)-cholesterol, low density lipoprotein (LDL)-cholesterol, aspartate aminotransferase (AST), alanine aminotransferase (ALT), γ-glutamyl transpeptidase (γ-GTP), total bilirubin, direct bilirubin, indirect bilirubin, alkaline phosphatase (ALP), blood urea nitrogen (BUN), creatinine, triglyceride, total protein, albumin, lactate dehydrogenase (LDH), and creatine kinase (CK).

### 2.5 Statistical plan

The participants’ characteristics will be summarized using means and standard deviations for continuous variables that satisfy normality or using median and interquartile range for non-normal data. Group differences will be assessed using 1-way analysis of variance (ANOVA) or the Kruskal–Wallis test. In addition, the independent-samples t-test or chi-squared test will be used to analyze differences between the AD and non-AD groups, as well as between the obese and non-obese groups.

The microbiome sequencing data obtained from feces will be analyzed using the Quantitative Insights into Microbial Ecology (QIIME) and assigned to an operational taxonomic units (OTU) table generated using the EzTaxon database. The Phylogenic Investigation of Communities by Reconstruction of Unobserved States (PICRUSt) program will be used to compare differences in gene expression. Alpha diversity metrics include the number of OTUs (species richness), the Simpson or Shannon diversity indices, and the Chao1 metric. Beta diversity will be analyzed using both unweighted and weighted UniFrac distances.

Metabolome data will be analyzed using univariate statistics and multivariate modeling. All analyses will be implemented in R statistical programming. Statistical differences among groups will be independently evaluated for known and unknown metabolites, and cluster analysis will be used to compare the metabolites profiles. Metabolites displaying significantly altered delta values (*P* <.05) will be used for principal components analysis (unsupervised) and orthogonal partial least squares analysis (supervised) projection to latent structures.

### 2.6 Discontinuation and drop-out criteria

The following are the criteria for discontinuation from the study:

(1) withdrawal of consent to participation in the study by the subject or their legal representative;
(2) discovery of violation of inclusion criteria or presence of exclusion criteria during the course of research;
(3) loss to follow-up;
(4) judgement of unsuitability for continuation in the study based on the discretion of the investigator in charge.

### 2.7 Ethics

The study protocol has been approved by the Institutional Review Board of Wonkwang University Iksan Korean Medicine Hospital (WKUIOMH-IRB-2018-05) and has been registered in the Clinical Research Information Service, Republic of Korea (KCT0003630). It accords with the Declaration of Helsinki. Written informed consent will be obtained from the participants and the legal representatives before enrolment in the study. If the subject is under 7 years old, written informed consent will only be obtained from the legal representatives.

## 3 Discussion

This is a study protocol to investigate the association of AD with obesity in the Korean population and verify its mechanism by analyzing gut microbiome–metabolome–immune biomarkers using a multi-omics approach.

In this study, we included 4 to 12-year-old children as subjects for several reasons. First, AD is one of the most common chronic childhood skin diseases, affecting up to 15% to 20% of children worldwide.^[16,17]^ Given that the symptoms of AD usually begin in infancy, the majority of AD cases develop before the age of 5 years,^[18,19]^ and the prevalence in teenagers seems to be falling significantly,^[20]^ children aged 4 to 12 have a high prevalence of AD. Second, most children aged 4 to 12 years in Korea, spend most of their time in a group setting, such as a kindergarten or school, eating a similar Korean-style diet consisting of rice, soup, and side dishes. Third, it is known that the phylogenetic composition of bacterial communities evolves towards an adult-like composition within 3 years after birth.^[21]^

In the present study, a 3-day controlled diet will be provided to ensure better dietary control, and fecal samples will be collected both before and after controlled meals. It is now understood that diet plays a significant role in shaping the microbiome, with experiments showing that dietary alterations can induce large, temporary microbial shifts within 24 hours.^[22,23]^ Because there is no appropriate guideline in terms of dietary control in microbiome analysis, we assumed that 3-day-controlled meal is sufficient to eliminate effects of diet. We also will analyze microbial changes before and after the 3-day meal to confirm whether the 3-day controlled meal is sufficient to remove microbial changes due to diet.

This study protocol represents a unique opportunity to enhance our understanding of the association between AD and obesity in terms of gut microbiome, metabolome, and immunological mechanisms and may, therefore, serve to improve future management strategies for AD.

## Acknowledgment

The authors thank So Young Jung and Bo-Young Kim at the Korea Institute of Oriental Medicine for monitoring study and developing electronic case report form.

## Author contributions

**Analysis:** Jeeyoun Jung, Mi Ju Son

**Conceptualization:** Jeeyoun Jung, Mi Ju Son

**Formal analysis:** Mi Ju Son, Jeeyoun Jung.

**Funding acquisition:** Jeeyoun Jung.

**Investigation:** Geum-Jin Yang, Yu-Hwa Shim, Su-Jin Kang, Ji-Eun Hong, Jung-Eun Lee, Jaemoo Chun, Seonghwan Park.

**Methodology:** Eun-Heui Jo, Young-Eun Kim

**Project administration:** Min-Cheol Park.

**Supervision:** Jeeyoun Jung, Min-Cheol Park.

**Writing – original draft:** Geum-Jin Yang, Mi Ju Son.

**Writing – review & editing:** Eun-Heui Jo, Yu-Hwa Shim, Su-Jin Kang, Ji-Eun Hong, Young-Eun Kim, Jung-Eun Lee, Jaemoo Chun, Seonghwan Park, Jeeyoun Jung, Min-Cheol Park.
